# An *in vitro*-identified high-affinity nucleosome-positioning signal is capable of transiently positioning a nucleosome *in vivo*

**DOI:** 10.1186/1756-8935-3-13

**Published:** 2010-07-01

**Authors:** Lia E Gracey, Zhi-Ying Chen, Jay M Maniar, Anton Valouev, Arend Sidow, Mark A Kay, Andrew Z Fire

**Affiliations:** 1 Department of Genetics, Stanford University School of Medicine, Stanford, CA, USA; 2 Department of Pediatrics, Stanford University School of Medicine, Stanford, CA, USA; 3 Department of Pathology, Stanford University School of Medicine, Stanford, CA, USA

## Abstract

**Background:**

The physiological function of eukaryotic DNA occurs in the context of nucleosomal arrays that can expose or obscure defined segments of the genome. Certain DNA sequences are capable of strongly positioning a nucleosome *in vitro*, suggesting the possibility that favorable intrinsic signals might reproducibly structure chromatin segments. As high-throughput sequencing analyses of nucleosome coverage *in vitro *and *in vivo *have become possible, a vigorous debate has arisen over the degree to which intrinsic DNA:nucleosome affinities orchestrate the *in vivo *positions of nucleosomes, thereby controlling physical accessibility of specific sequences in DNA.

**Results:**

We describe here the *in vivo *consequences of placing a synthetic high-affinity nucleosome-positioning signal, the 601 sequence, into a DNA plasmid vector in mice. Strikingly, the 601 sequence was sufficient to position nucleosomes during an early phase after introduction of the DNA into the mice (when the plasmid vector transgene was active). This positioning capability was transient, with a loss of strong positioning at a later time point when the transgenes had become silent.

**Conclusions:**

These results demonstrate an ability of DNA sequences selected solely for nucleosome affinity to organize chromatin *in vivo*, and the ability of other mechanisms to overcome these interactions in a dynamic nuclear environment.

## Background

Enzymes that interact with DNA to direct transcription, replication and repair are dependent on physical accessibility of the sequences to which they can initially bind. At any given time, the majority of DNA sequences in a eukaryotic nucleus are tightly wrapped around proteinaceous histones, forming nucleosome cores [1]. On a structural level, the stereotypic patterns of nucleosome spacing provide a first layer in the three-dimensional organization of chromosomes [2]. On a functional level, the nucleosome landscape consists of relatively accessible 0-80 base internucleosome linker regions interspersed between the nearly inaccessible nucleosome cores (146 to 147 bases each) [3]. Thus, a central focus in studying gene regulation is based on understanding how the dynamic interactions of DNA with histones and other proteins contribute to the precise register and pitch of genomic chromatin in key chromosomal regions.

A variety of naturally occurring and synthetic DNA sequences have been shown to be sufficient for positioning a nucleosome in purified *in vitro *reconstitution systems [4-6]. The genomic positions of nucleosomes have also been extensively documented from several systems *in vivo*, again indicating nonrandom association of nucleosome positions with specific sequence features in DNA in certain areas of the genome [7]. Despite intensive analyses of the statistical correspondence between *in vitro *positioning capability and *in vivo *nucleosome positions, there is a lack of consensus on the degree to which the physiological nucleosome landscape is specified by intrinsic DNA:nucleosome affinities [8-12], and a lack of data addressing the ability of biochemically-identified nucleosome-positioning sequences to control *in vivo *nucleosome occupancy over time.

## Results and Discussion

### *In vivo *assays for the function of a synthetic nucleosome-positioning signal in a mouse model system

**Figure 1:**
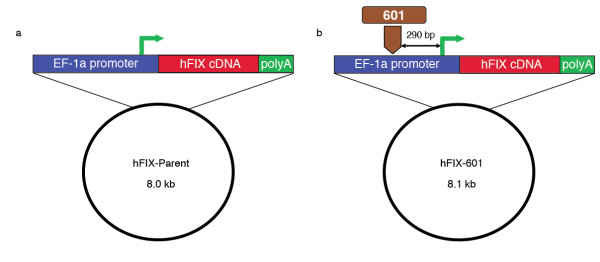
**Insertion of the 601 nucleosome positioning sequence upstream of the transcription start site in a human factor IX (hFIX) expression vector**. Bent green arrow denotes the transcription start site. **(a) **hFIX-Parent vector. **(b) **hFIX-601 vector.

The synthetic 601 sequence [5], derived by Lowary and Widom solely based on its affinity and ability to position nucleosomes, has been a reliable standard positioning signal in numerous *in vitro *studies [e.g., [13-17]]. The ability of 601 to position a nucleosome *in vivo *is an open question that is of considerable interest. To study the *in vivo *positioning capability of the 601 sequence, we used a parent vector with a human Factor IX (hFIX) cDNA gene driven by the human elongation factor-1 alpha (EF-1α) promoter (Figure 1a, hFIX-Parent). The 601 sequence was inserted within the promoter region upstream of the transcription start site (TSS) (Figure 1b, hFIX-601).

A mouse gene transfer system was used to test the dynamics of the strong 601 *in vitro *positioning signal in defining the location of a nucleosome *in vivo*. The hFIX constructs were delivered to 6- to 8-week-old C57/Bl6 female mice by hydrodynamic tail vein injection. With this method, the injected naked plasmid DNA is preferentially taken up by hepatocytes in the liver, exists in an episomal form, and may be present in more than one copy in any individual cell [18-20]. This system has been used to quantify the corresponding transgene expression products made from various delivered DNA molecules in a functional tissue over time. It has consistently been observed in such assays that exogenous transgenes in bacterial plasmid-based vectors are efficiently silenced within the first several weeks after injection, even though the DNA is still present in the nucleus of the hepatocytes [20].

To measure transgene expression after DNA injection, we quantified the serum hFIX protein over time. As expected, the infusion of hFIX-Parent and hFIX-601 resulted in hFIX levels that peaked within the first 3 days and then dropped by over 80-90% in a period of 4-6 weeks (see Additional file 1, Figure S1).

For the analyses of *in vivo *chromatin structures, we harvested the transfected mouse livers at 3 days and 6 weeks to represent either high or silenced levels of hFIX expression, respectively. Micrococcal nuclease (MNase) was used as a probe for nucleosome positions [21]. The digested DNA was size-selected for mononucleosome core DNA (average fragment size approximately 150 bp, including both mouse and vector-specific sequences), ligated to linkers compatible with the Illumina/Genome Analyzer II (GAII) high-throughput sequencing platform, and selected by hybridization to biotin-tagged single-stranded DNA probes. After washing and boiling, the hybridized DNA was amplified and sequenced using the Illumina/GAII platform.

### Positioning of nucleosomes over the 601 sequence after hydrodynamic injection

**Figure 2:**
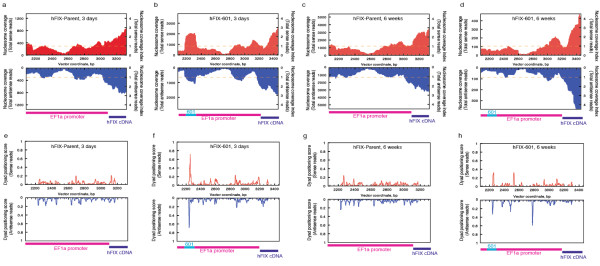
**Nucleosome coverage and dyad positioning scores for nucleosomes in the promoter of human Factor IX (hFIX) constructs hFIX-Parent and hFIX-601 during time points of high expression and silencing**. The dashed line at index = 1 represents the average expected nucleosome coverage at a given base pair. Vector base pair coordinate is plotted on the X-axis. Colored bars below each graph represent the span of the labeled vector DNA element. **(a) **Nucleosome coverage in the hFIX-Parent promoter at 3 days. **(b) **Nucleosome coverage in the hFIX-601 promoter at 3 days. **(c) **Nucleosome coverage in the hFIX-Parent promoter at 6 weeks. **(d) **Nucleosome coverage in the hFIX-601 promoter at 6 weeks. **(e) **Dyad positioning scores in the hFIX-Parent promoter at 3 days. **(f) **Dyad positioning scores in the hFIX-601 promoter at 3 days. **(g) **Dyad positioning scores in the hFIX-Parent promoter at 6 weeks. **(h) **Dyad positioning scores in the hFIX-601 promoter at 6 weeks.

Various measures of nucleosome occupancy and positioning from high-throughput sequencing data have been used in recent studies, serving as a basis for an expanding and lively ongoing discussion of chromatin organization [e.g. [12,22-26]]. We applied many of these measures to the data (Figure 2; see Additional file 1, Figure S2, Figure S3, Figure S4). For the following discussion, we have chosen two representations - coverage plots [12] (Figure 2a-d) and dyad positioning scores [26] (Figure 2e-h) - to display nucleosome patterns. For additional representations of the data, see Additional file 1 (Figure S2, Figure S3, Figure S4).

Nucleosome coverage plots [12] display the number of overlapping nucleosomes observed at each base in the vector (counts on the left axis). These graphs also show the normalized nucleosome coverage index (right axis), which was calculated by normalizing to the average for the entire assayed DNA segment. The dashed line at index = 1 represents the average expected nucleosome coverage.

Dyad positioning scores [25] were obtained by counting the number of nucleosome dyads that sit within a fixed narrow window (± 5 bp) around a particular base pair, and dividing this by the number of dyads in a larger window (± 150 bp). We defined the dyad (middle) of the nucleosome by using the start of the Illumina/GAII sequence read and adding 75 bp. The inferred 75 bp start-to-dyad offset is based on the average DNA fragment size of 150 bp. It is known that the precise position of final MNase cleavage and trimming in any individual chromatin fragment relative to the actual nucleosome core is a function of sequence and stochastic events during digestion [27,28]. The ± 5 bp window provides a picture in which nucleosome positioning is accentuated relative to the noise from imprecision in MNase trimming. The resulting dyad positioning score will be 1.0 at sites where a nucleosome is uniquely and universally positioned, 0.0 at sites where a dyad is never observed and 11/301 (0.037) in areas where dyad positioning is completely uniform without any apparent sequence preference. Dyad positioning scores were independently calculated for the reads obtained from the + and - strands. Although we occasionally observed spurious peaks of high dyad positioning scores in one strand, we defined a true positioning signal as one that had peaks occurring in both the + and - strands.

As MNase was used as the major tool to examine nucleosome coverage, it was important to examine the spectra of MNase cleavage sites on supercoiled naked DNA. Experimental MNase cleavage of naked DNA with low concentrations of enzyme can produce fragments comparable in size to nucleosomes. When these DNAs were generated from our circular naked plasmids (with or without selective hybridization of plasmid sequences), we observed a nonuniform pattern of coverage (see Additional file 1, Figure S5); similar results have been observed in high-throughput sequencing studies of genomic DNA [24]. Of particular interest in this study is the appearance of cleavage sites near the termini of 601 on naked hFIX-601 (see Additional file 1, Figure S5d). These sites are certainly not unique; numerous other sites in this area and in the encompassing vector are also observed from naked DNA cleavage and capture. This is the background on which we then examined the *in vivo *structures of chromatin templates.

For the hFIX-Parent vector (without 601), most positions showed flexibility in nucleosome dyad positioning. Few examples of nucleosome constraint (peaks) were observed, and dyad positioning scores for the bulk of plasmid sequences fell under 0.20, indicating that most sequences failed to exert strong positioning in this context (Figure 2e, g; see Additional file 1, Figure S4a, b). We also noted that some (but not all) of the peaks in the dyad positioning analysis were shared with naked DNA (see Additional file 1, Figure S5a), indicating that in this range of apparent positioning scores, MNase biases may coexist with real (but modest) *in vivo *positional constraint. The hFIX-Parent vector did not show any substantial changes in nucleosome coverage between the 3-day and 6-week liver samples (Figure 2a, c; see Additional file 1, Figure S4a, b).

The 601 sequence produced a very different positioning pattern in comparison to any site in the hFIX-Parent vector. At the 3-day time point, 70-80% of impinging nucleosomes for this segment (dyad positioning score 0.70-0.80; Figure 2f) were localized within 1-10 bp of the *in vitro *601 dyad, previously determined by hydroxyl radical footprinting [29]. Precise positioning of nucleosomes at the single base level is challenging, given the well-documented biases of MNase cleavage [27,28]. Indeed, the precise cleavages that we observed at the ends of the 601 segment were also observed at some level as limited local cleavage maxima in the naked DNA experiments (see Additional file 1, Figure S5d) with somewhat stronger local preference on the - than the + strand. This is consistent with intrinsic MNase preferences determining the precise *in vivo *fragment ends in a narrow accessible region adjacent to (or just within) each nucleosome [30]. Additional features of the profile are evident from an alternative estimate of position using a weighted kernel density calculation (still subject to local MNase preference uncertainty) that gave a preferred dyad position at base pair 2250 (Methods; see Additional file 1, Figure S2b), within 8 bp of the *in vitro *601 dyad. Coverage plots show the overall nucleosomal landscape, with higher nucleosome coverage over the 601 sequence (Figure 2b; see Additional file 1, Figure S5e, which shows a similar profile when *in vivo *counts were normalized to MNase-treated naked DNA read counts).

### A decrease in measured nucleosome positioning over 601 accompanies transgene silencing

We observed a loss of nucleosome coverage over the 601 sequence at 6 weeks, when expression had been silenced (Figure 2d). The dyad positioning scores at the 601 sequence dropped to approximately 0.35, whereas nearby positions had scores that increased from 0.10-0.15 to 0.20-0.40 (Figure 2h). This result highlights a dynamic process in which a transient preference of a nucleosome for occupying a thermodynamically favorable sequence is converted over time to an alternative chromatin conformation.

It is possible that the conversion between patterns results from a shift in higher-order structure in which the resulting structure remains chromatinized but is subject to forces that restrict the ability of 601 to position nucleosomes. As an alternative, we also considered the possibility that the 6-week samples might reflect a non-chromatin state of the injected DNA. Several lines of evidence support the existence of a chromatin structure on hydrodynamically delivered plasmids at late post-injection time points in the mouse liver. First, using immunoprecipitation with antibodies to modified and canonical histone proteins, Riu *et al.*[31] found and analyzed histone marks present on a comparable set of circular DNAs after the same method of hydrodynamic delivery. The plasmids had a different expression cassette (Rous sarcoma virus promoter driving human α1-antitrypsin), but were of similar size and had the same bacterial backbone.

Second, *in situ *hybridization analysis of livers from hydrodynamically injected mice [32] showed that the majority of plasmid DNA remains in the nucleus at 2 to 3 weeks after the standard post-injection drop in expression level (see Additional file 1, Figure S1).

Third, we found that vector DNA fragments that were captured by selective hybridization, sequenced by the Sanger method and aligned to the vector were of the approximate length of a nucleosome core DNA at both the 3-day (mean ± SD 146 ± 14.4 bp) and 6-week (146 ± 16.0 bp) time points. These data support the hypothesis that the injected DNA remains nuclear and in a predominantly nucleosomal context, even as expression has been silenced weeks after injection.

### Additional aspects of observed nucleosome coverage and dyad positioning

The various nucleosome coverage and dyad positioning plots suggest several additional intriguing features of the overall nucleosome landscape over the injected vector DNA. Such features, and indeed any specific aspect of the pattern, could be reflective of real differences or be due to technical differences in the liberation, capture and sequencing of the nucleosomal DNA fragments.

Some regions of low nucleosome coverage in the *in vivo *plots may result from sequences with a high GC content, which are intrinsically resistant to MNase digestion. For example, one such area may occur close to base pair 2700 (76% GC content) (Figure 2), especially as a similar, apparent nucleosome-free region was found via naked DNA digest by MNase (see Additional file 1, Figure S5c). This property would be consistent with previously observed MNase cutting biases [27,28]. Although we have supporting evidence that the plasmid is wrapped in nucleosomes, we cannot guarantee that the structure observes the same rules as genomic chromosomal DNA, given the episomal circular nature of the plasmid. Thus, the best estimate we can make based on an MNase digestion is a relative estimate of nucleosome abundance, not an absolute measure.

## Conclusions

In summary, we observed conditional activity of a strong nucleosome-positioning element *in vivo *(in mouse liver) after hydrodynamic delivery of DNA. Shortly after the introduction of foreign DNA, when the DNA is active, the 601 positioning signal is capable of consistently positioning a nucleosome within a few base pairs on the underlying DNA. As the exogenous transgene silences, other forces govern nucleosome positioning in the region, and the tight localization is lost. It will be of future interest to study the positioning capability of synthetic high-affinity nucleosome positioning signals in other contexts and model systems.

In conclusion, these studies illuminate (i) a significant but not absolute relationship between DNA affinity and *in vivo *nucleosome-positioning, previously observed in isolated *in vitro *systems; (ii) the additional complex positioning influences in chromatin that respond to biological regulation processes; and (iii) the possibility of dictating the early post-delivery nucleosomal landscape (and thereby accessibility for the transcriptional machinery) for future therapeutic DNA vectors.

## Methods

### Vector construction

One copy of the 601 sequence was added to a previously described factor IX expression vector [33] upstream of the annotated transcription start site. Plasmid DNA was prepared as described [33]. The sequence of the insertion and flanking regions is (cctaggaatGCCCTGGAGAATCCCGGT*CTGC*AGGCCGCTCAATTGGTCGTAGACAGCTCTAGCACCGCTTAAACGCACGTACGCGCTGTCCCCCGCG TTTTAACCGCCAAGGGGATTACTCCCTAGTCTCCAGGCACGTGTCAGATATATACATCCTGTGCAggtccatgg) with the original cloning vector sequences in lowercase, synthetic inserted DNA in uppercase, and italic letters denoting differences from the original 601 sequence [5,16] based on Sanger sequencing of the 601 clone that was used.

### Animal studies

All animal procedures were conducted in accordance with guidelines set by the National Institutes of Health, the Animal Welfare Act, and the Stanford University School of Medicine. All procedures were approved by the Institutional Animal Care and Use Committee under protocol #13545.

C57/Bl6 female mice at 6-8 weeks were used for all experiments. Using a hydrodynamic (high-volume) injection protocol [18,19], 20 ug of plasmid DNA in 0.9% saline were injected into the tail vein of the mice. Serum samples were collected at several time points after injection via retro-orbital bleeding for hFIX protein quantification by ELISA [34].

### Nucleosome core DNA isolation

Mouse livers were harvested at either 3 days or 6 weeks post-injection and flash-frozen in liquid nitrogen. Liver tissue was ground in liquid nitrogen and digested with 1000 U MNase at 16°C for 5 minutes. Mononucleosome core DNA was isolated using agarose gels [30], and ligated to linkers compatible with the Illumina/GAII high-throughput sequencing platform. PCR amplification was performed with primers complementary to the Illumina linkers.

### Selective hybridization

A biotin-labeled probe against a 1.5 kb portion of the vector was generated and mixed with the amplified Illumina-linkered nucleosome core DNA populations from mouse liver. After washing and boiling, the hybridized DNA was amplified by PCR for a minimal number of rounds and analyzed using a Genome Analyzer II (GAII) sequencing system (Illumina).

As a negative control, we isolated mononucleosome core DNA from a saline-only mock-transfected mouse liver. After incubating the amplified core DNA with a biotin-labeled probe from the vector, no significant population of sequences corresponding to the vector was pulled down (data not shown), thereby indicating specificity of the hybridization.

### Nucleosome coverage calculation

BLAT was used to align the Illumina/GAII sequence reads to the respective vector, and only perfect matches were used for further analysis. Coverage plots were generated by adding 147 bp to the first base pair of an Illumina/GAII read to represent the canonical nucleosome. Each base pair received a count of 1 if it fell within the extended nucleosome. Total counts were then added to represent the nucleosome density. Normalized nucleosome coverage index values were generated by dividing the number of sequence reads at a particular base pair by the average coverage for the probe (the total sequence reads for a particular probe divided by the length of the probe).

### Start and end density calculation

To generate start and end density plots, the number of Illumina/GAII reads that started at a given base pair in the sense (start) or antisense (end) orientation were counted. Counts were smoothed over a window of 11 bp. Normalized index values were generated by dividing the average number of sequence reads in an 11 bp window around a particular base pair by the average coverage for the probe (the total sequence reads for a particular probe divided by the length of the probe).

### Dyad positioning score calculation

Dyad positioning scores reflect the preference for a single nucleosome to sit in a fixed narrow window versus another nearby position. The dyad was defined by adding 75 bp to the start of the Illumina/GAII read or subtracting 75 bp from the end. The positioning score for a given vector base pair was calculated by counting the number of dyads in a narrow window (± 5 bp) around a particular base pair, and dividing by the number of dyads in a larger window (± 150 bp) around the same base pair. A correction factor was added for low coverage areas by subtracting the square root of the narrow window total from the narrow window total, and adding the square root of the large window total to the large window total.

### Nucleosome density and stringency plot generation

Nucleosome density plots represent non-normalized kernel density estimates of dyad distribution as previously described [35]. Nucleosome positioning stringency reflects the fraction of dyads within a region that falls within the 'well positioned' configuration. This metric is calculated by normalizing the dyad density estimate within a 300 bp window as previously described [35].

## Competing interests

The authors declare that they have no competing interests.

## Authors' contributions

LEG and AZF developed the experiments and analyzed and interpreted data. LEG performed the experiments. AS and MAK provided additional guidance in experimental design and interpretation of data. ZYC conceived of and carried out initial development of the selective hybridization method. JMM and AV carried out bioinformatic analyses of sequence data. LEG and AZF wrote the manuscript. All of the authors read and approved the manuscript.

## Supplementary Material

Additional file 1 **Supplementary material**. Supplementary figures S1 to S6. Click here for file
