# Supplementary material for: An in vitro-identified high-affinity nucleosome-positioning signal is capable of transiently positioning a nucleosome in vivo
Source: Epigenetics Chromatin. 2010 Jul 1;3:13. doi: 10.1186/1756-8935-3-13 (PMC2915997; doi:10.1186/1756-8935-3-13)
Supplement: Additional file 1 — Supplementary material. Supplementary figures S1 to S6. [file 1756-8935-3-13-S1.PDF]

Supplementary Material Figure Legends

Supplementary Figure 1

ELISA expression data shows levels of hFIX protein produced at multiple timepoints. Pattern of hFIX silencing over time observed in mice injected with either hFIX-Parent (blue) or hFIX-601 (red). **(a)** Injection group with individual mice used for experiments. hFIX levels at 6 weeks were too low to be measured accurately by ELISA for all but two mice. **(b)** Expression pattern in a separate injection group of mice.

Supplementary Figure 2

Nucleosome coverage, dyad positioning scores, and start and end density for nucleosomes in the promoter of hFIX-Parent and hFIX-601 during high expression and silencing. Vector basepair coordinate is plotted on the x-axis. Colored bars below each graph represent the span of the labeled vector DNA element.

(a) Dyad density and stringency scores in the hFIX-Parent promoter at 3 days.
(b) Dyad density and stringency scores in the hFIX-601 promoter at 3 days.
(c) Dyad density and stringency scores in the hFIX-Parent promoter at 6 weeks.
(d) Dyad density and stringency scores in the hFIX-601 promoter at 6 weeks.
(e) Start and end read density of the hFIX-Parent promoter at 3 days.
(f) Start and end read density of the hFIX-601 promoter at 3 days.
(g) Start and end read density of the hFIX-Parent promoter at 6 weeks.
(h) Start and end read density of the hFIX-601 promoter at 6 weeks.

Supplementary Figure 3

Characterization of additional hFIX-601 mice from the same injection group.

(a) Nucleosome coverage in the hFIX-601 promoter at 3 days.
(b) Nucleosome coverage in the hFIX-601 promoter at 6 weeks.
(c) Dyad positioning scores in the hFIX-601 promoter at 3 days.
(d) Dyad positioning scores in the hFIX-601 promoter at 6 weeks.
(e) Dyad density and stringency scores for the hFIX-601 promoter at 3 days.
(f) Dyad density and stringency scores for the hFIX-601 promoter at 6 weeks.

Supplementary Figure 4

Nucleosome coverage, dyad positioning scores, and dyad density and stringency scores around the vectors outside of the promoter region. Vector basepair coordinate is plotted on the x-axis. Each panel i.-v. (or vi.) represents the data for a different probe around the vector. Colored bars below each graph represent the span of the labeled vector DNA element. LacZ N' = N-terminus fragment of LacZ coding region in vector backbone. 5' Flank = genomic flanking region of the EF-1α promoter. hGH poly A = human Growth Hormone poly-A signal. AmpR = Ampicillin resistance gene.

(a) hFIX-Parent at 3 days. **(b)** hFIX-Parent at 6 weeks. **(c)** hFIX-601 at 3 days. **(d)** hFIX-601 at 6 weeks.

Supplementary Figure 5

Comparisons of DNA fragments resulting from (i) heavy MNase digest of *in vivo* hFIX-Parent and hFIX-601 in mouse liver extracts or (ii) light MNase digest of naked hFIX-Parent and hFIX-601 DNA. Vector basepair coordinate is plotted on the x-axis. Colored bars below each graph represent the span of the labeled vector DNA element.

- **(a)** Dyad positioning scores in the hFIX-Parent promoter for naked hFIX-Parent DNA (top panels) and *in vivo* hFIX-Parent (bottom panels reproduced from Figure 2e).
- **(b)** Dyad positioning scores in the hFIX-601 promoter for naked hFIX-601 DNA (top panels), naked hFIX-601 DNA fragments with selective hybridization (middle panels), and *in vivo* hFIX-601(bottom panels reproduced from Figure 2f).
- **(c)** Nucleosome coverage in the hFIX-601 promoter for naked hFIX-601 DNA (top panels), naked hFIX-601 DNA fragments with selective hybridization (middle panels), and *in vivo* hFIX-601 (bottom panels reproduced from Figure 2b).
- **(d)** Smoothed start and end read density of the hFIX-601 promoter for naked hFIX-601 DNA (top panels), naked hFIX-601 DNA fragments with selective hybridization (middle panels), and *in vivo* hFIX-601 (bottom panels reproduced from Figure S2f)
- **(e)** Nucleosome coverage in the hFIX-601 promoter of *in vivo* hFIX-601 (with reads normalized to the average nucleosome coverage) divided by the nucleosome coverage of naked hFIX-601 DNA (with reads normalized to the average nucleosome coverage).
- **(f)** Raw, unsmoothed start and end read density tiled across hFIX-601 from heavy MNase digest of *in vivo* hFIX-601 (upper panels) and light digest of naked hFIX-601 (lower panels).

Supplementary Figure 6

5’ RACE results indicate an alternate transcription start site (TSS). (a) Annotation of the EF-1α promoter sequence. Orange arrowhead represents the annotated TSS of human EF-1α in human leukemia HL-60 cell culture [1]. Green arrow is the 5’ RACE-identified TSS of human EF-1α (in hFIX-601) in mouse liver at 3 days. **(b)** Representation of the alternate TSS relative to DNA elements in the vector. Reproduced from Fig. 2b. Orange arrowhead represents the annotated TSS of human EF-1α in human leukemia HL-60 cell culture [1]. Green arrow is the 5’ RACE-identified TSS of human EF-1α (in hFIX-601) in mouse liver at 3 days.

1. Uetsuki T, Naito A, Nagata S, Kaziro Y: **Isolation and characterization of the human chromosomal gene for polypeptide chain elongation factor-1 alpha.** *J Biol Chem* 1989, **264**:5791-8.

a

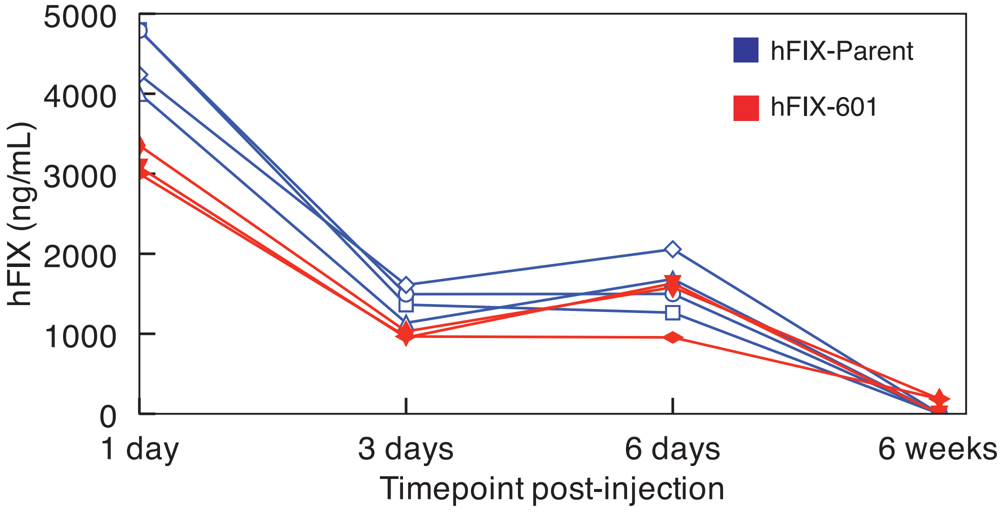

b

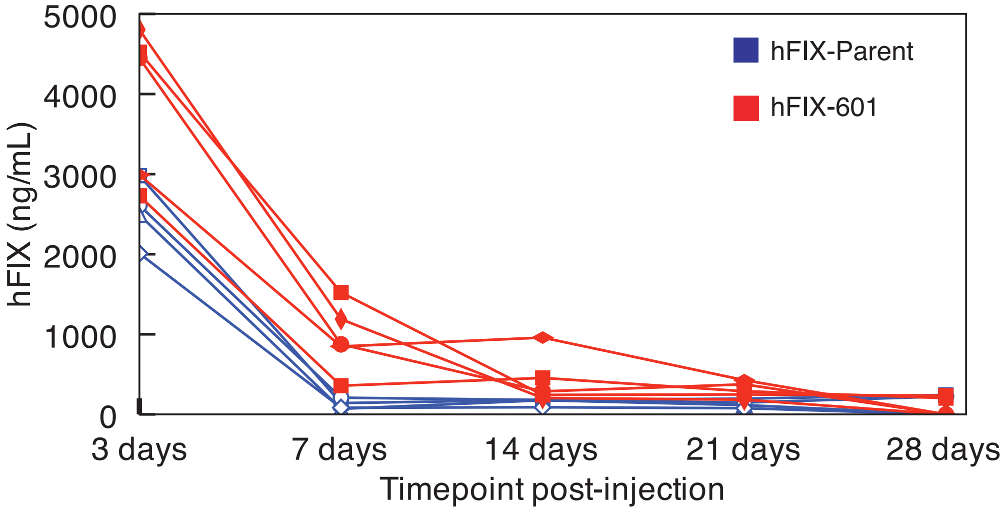

Supplementary Figure 1

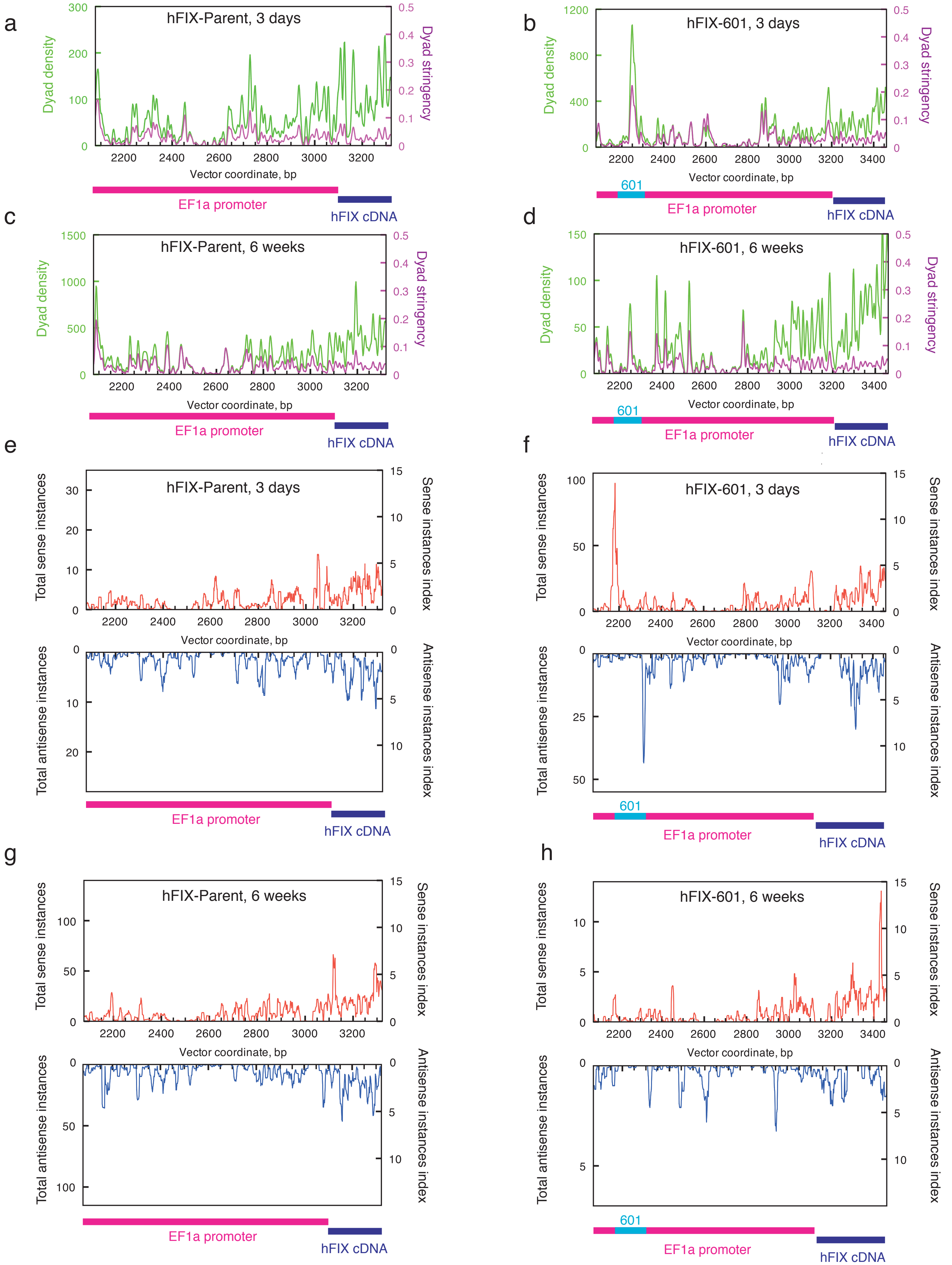

Supplementary Figure 2

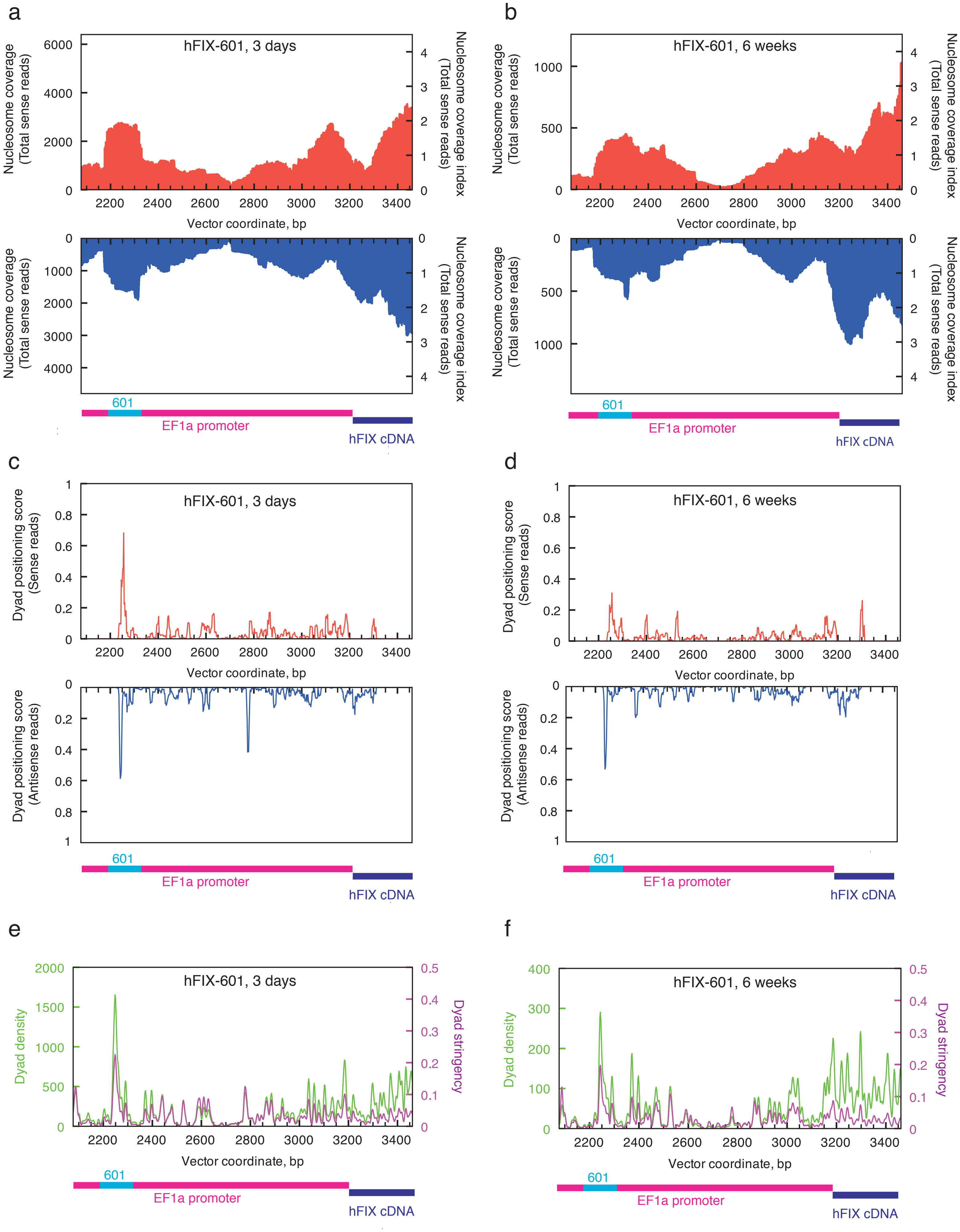

Supplementary Figure 3

Text with Supplementary Figure 4

In regions of the hFIX transgene outside of the promoter region, the chromatin landscape largely reflected a flexibility for nucleosome positions throughout the transgene. There appeared to be a nucleosome free region (NFR) at the 3’ end of the hFIX transgene in all vectors (Figure S4).

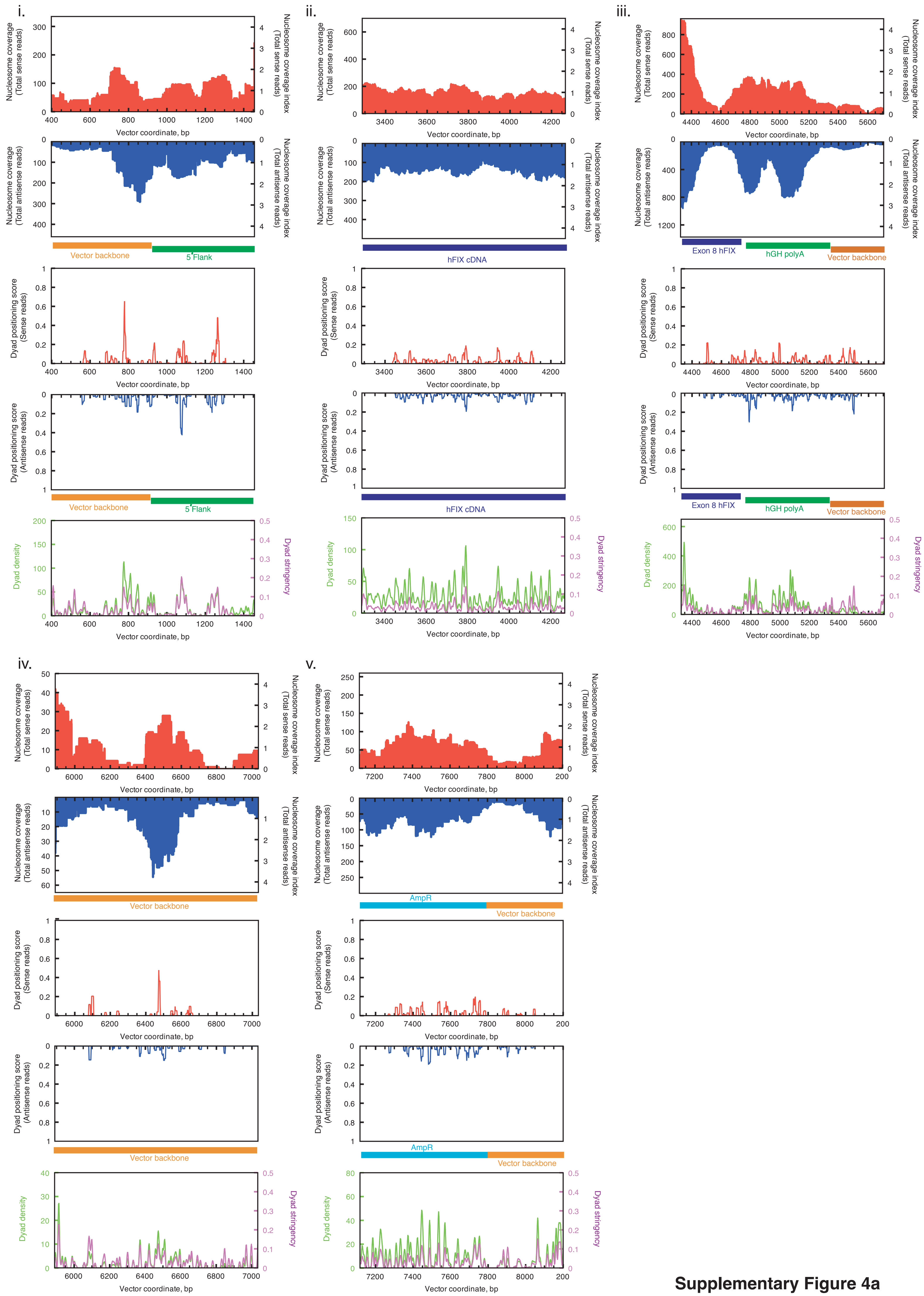

Supplementary Figure 4a

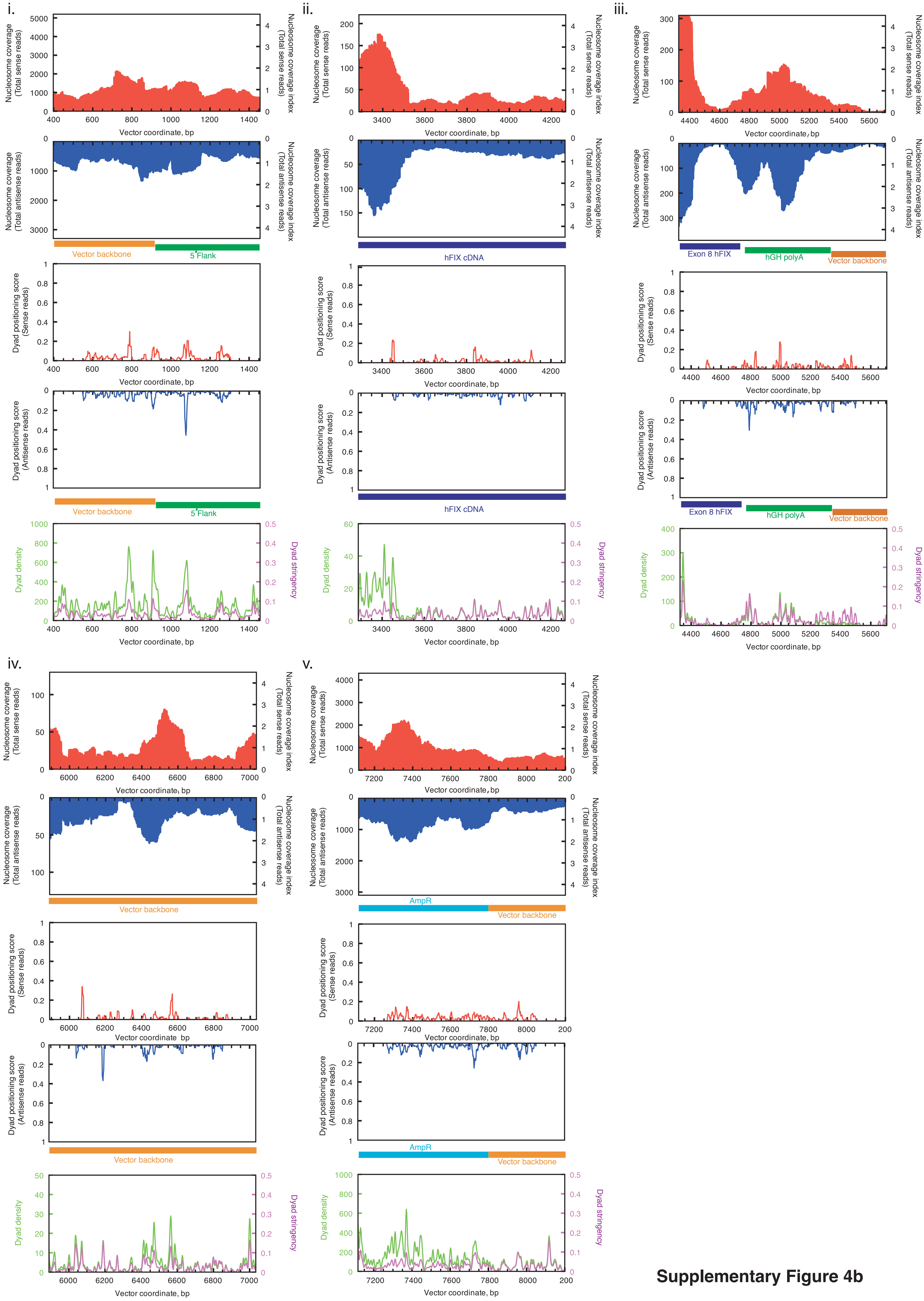

Supplementary Figure 4b

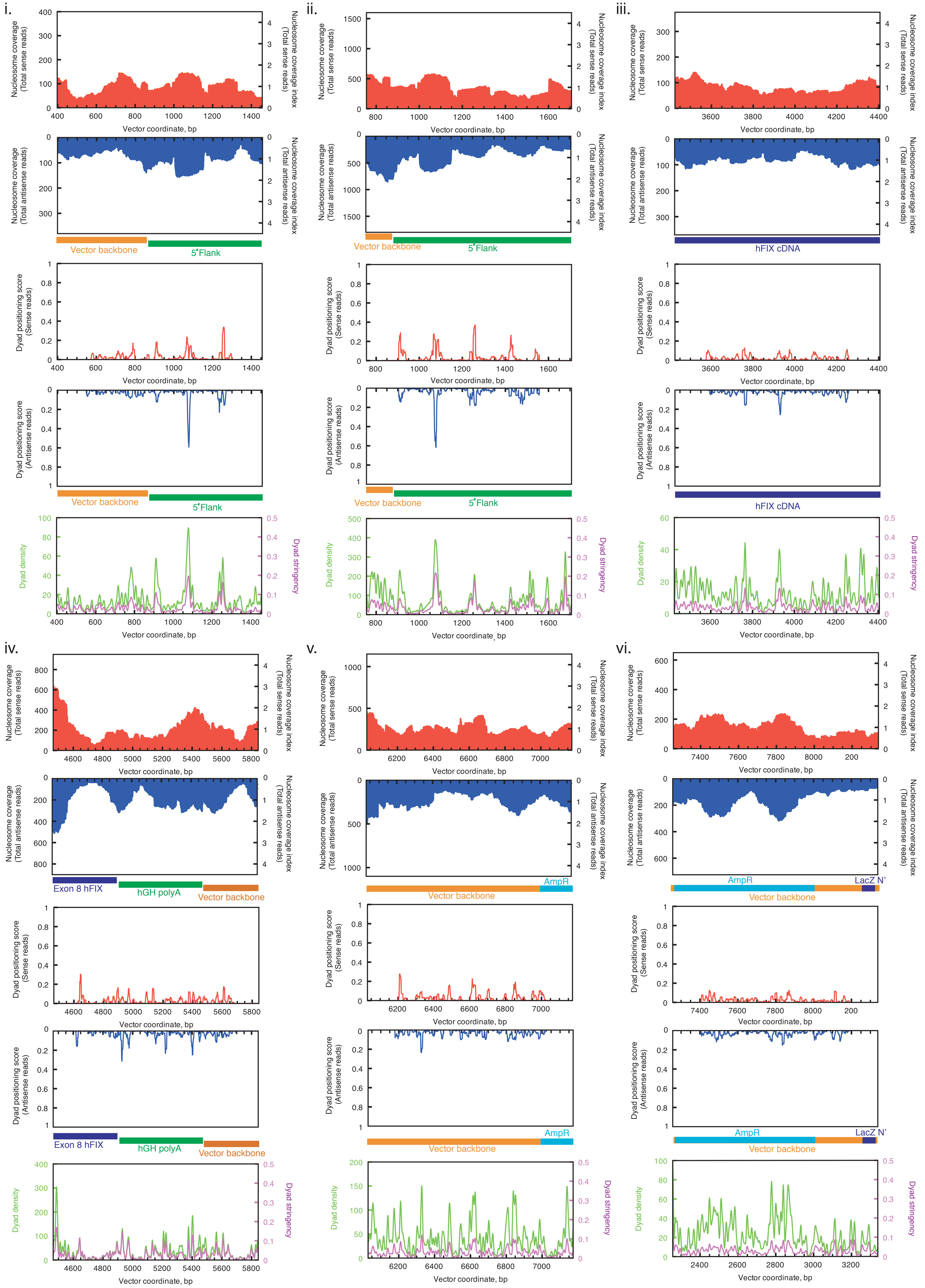

Supplementary Figure 4c

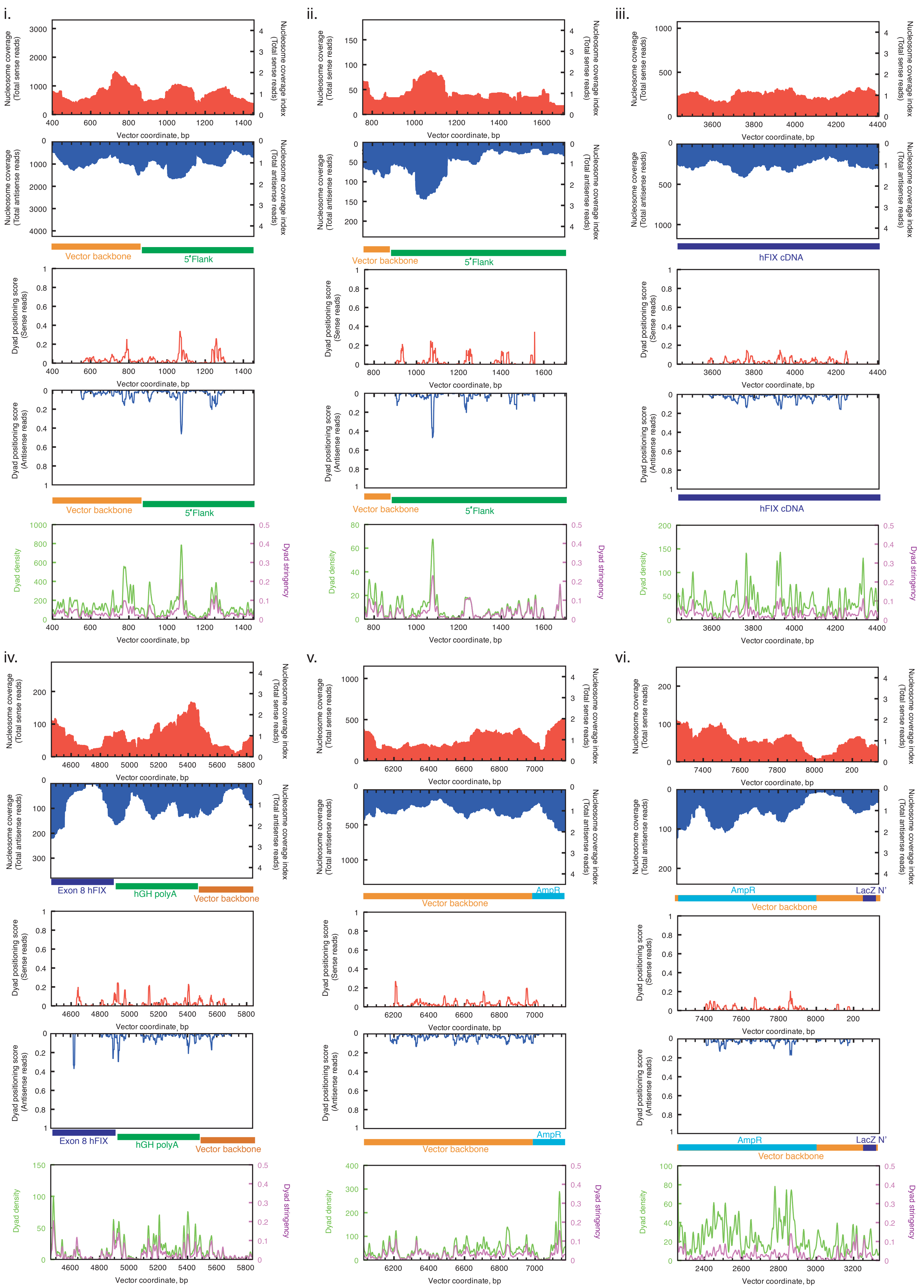

Supplementary Figure 4d

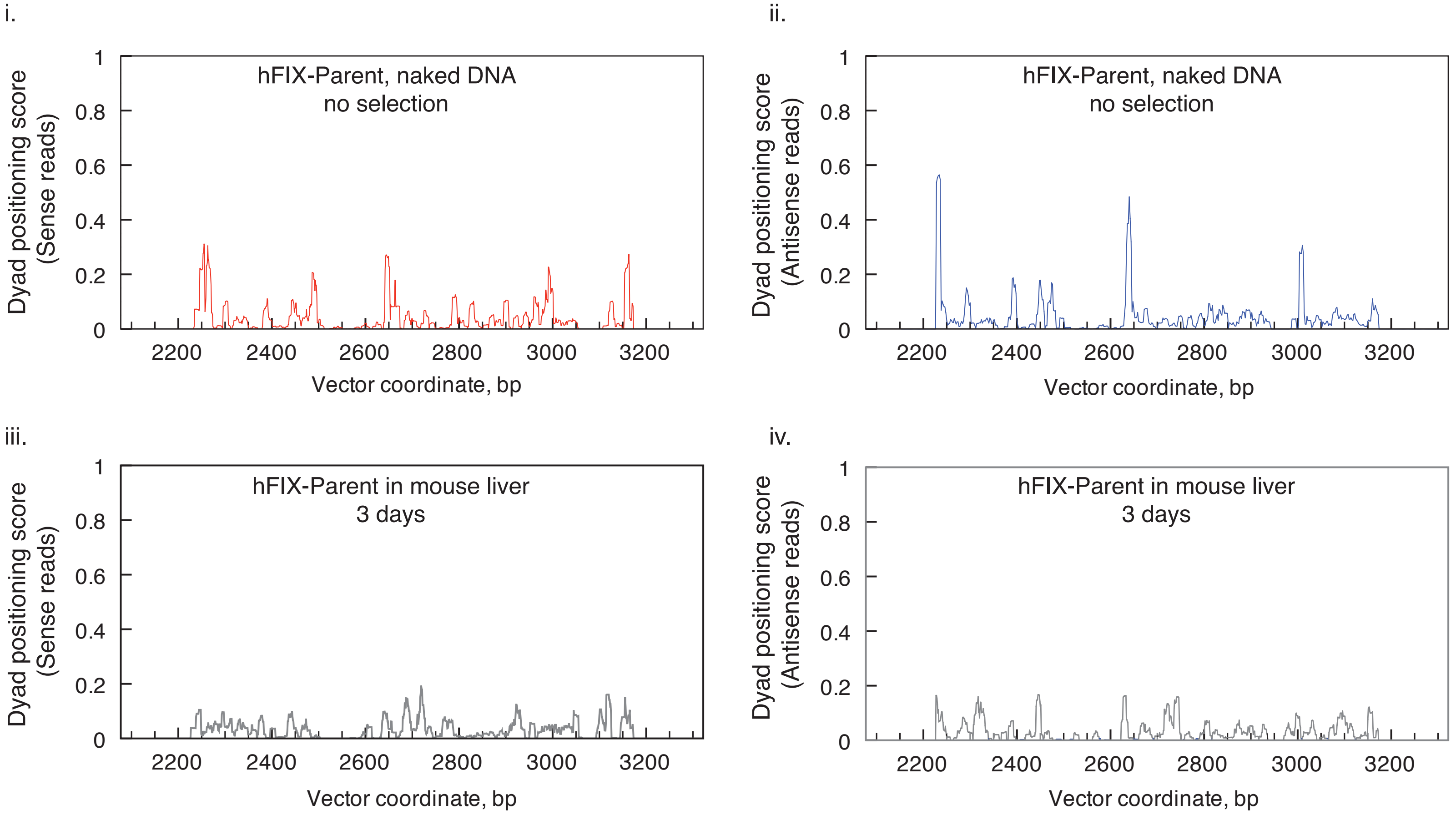

Supplementary Figure 5a

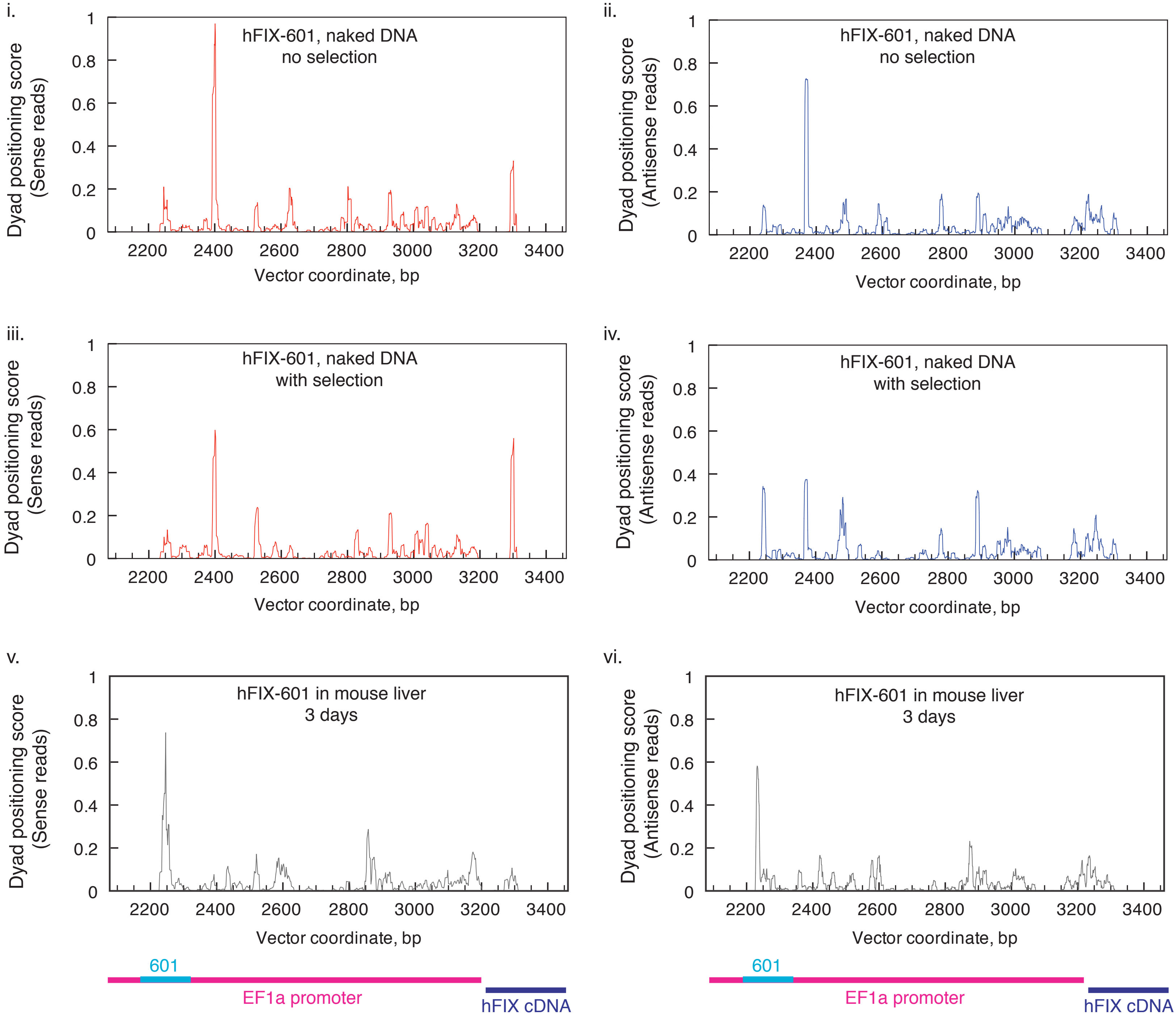

Supplementary Figure 5b

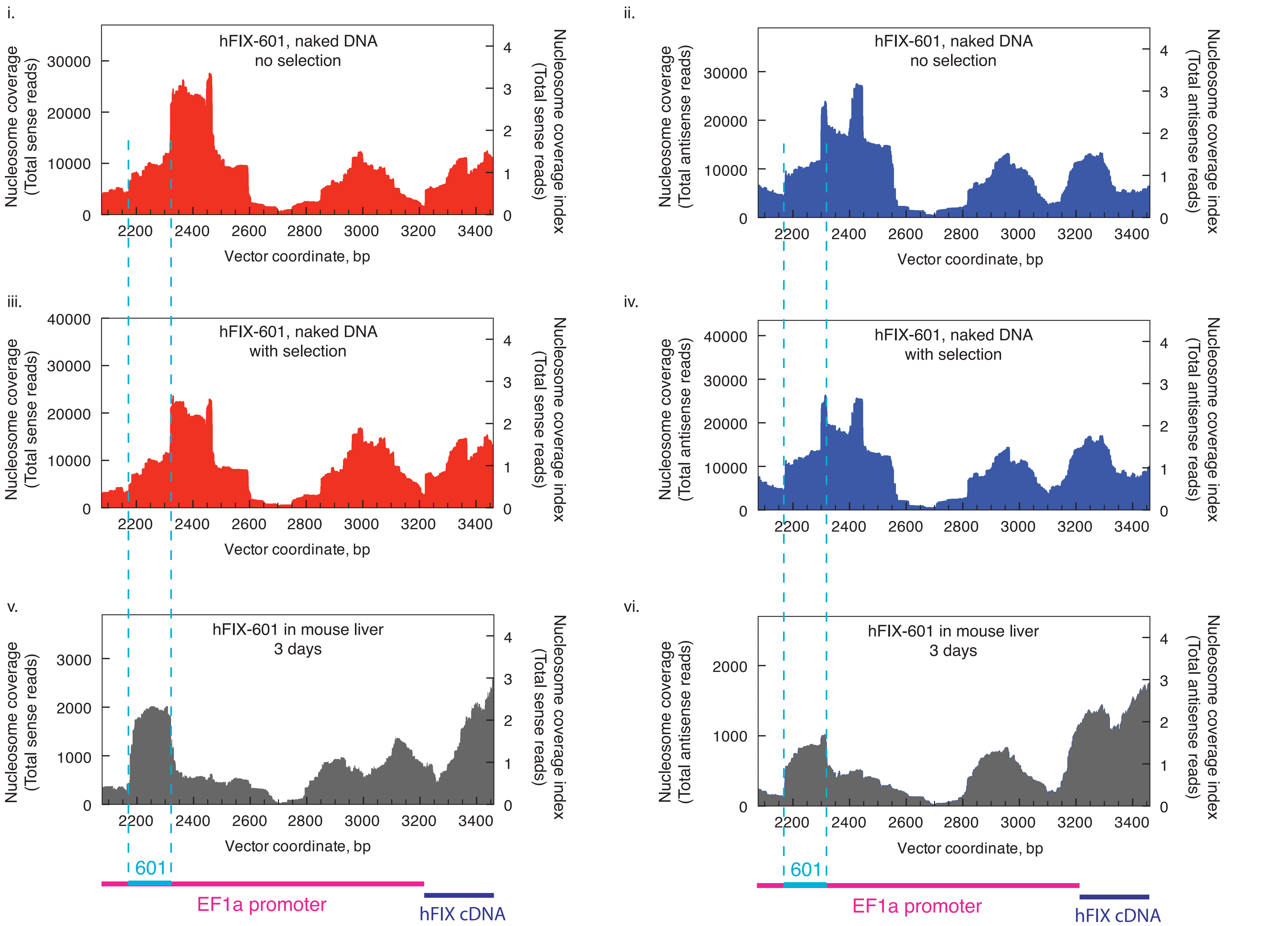

Supplementary Figure 5c

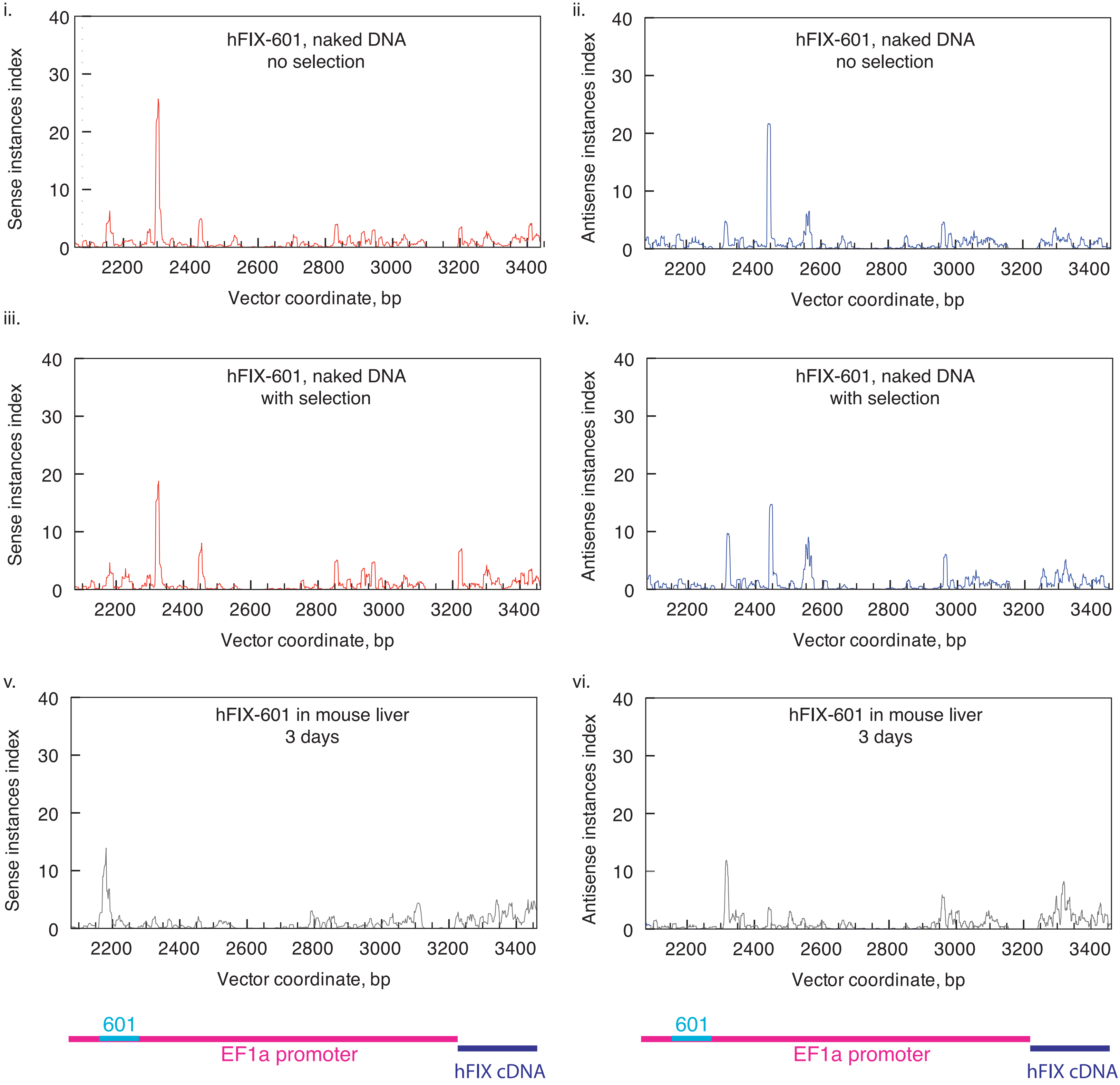

Supplementary Figure 5d

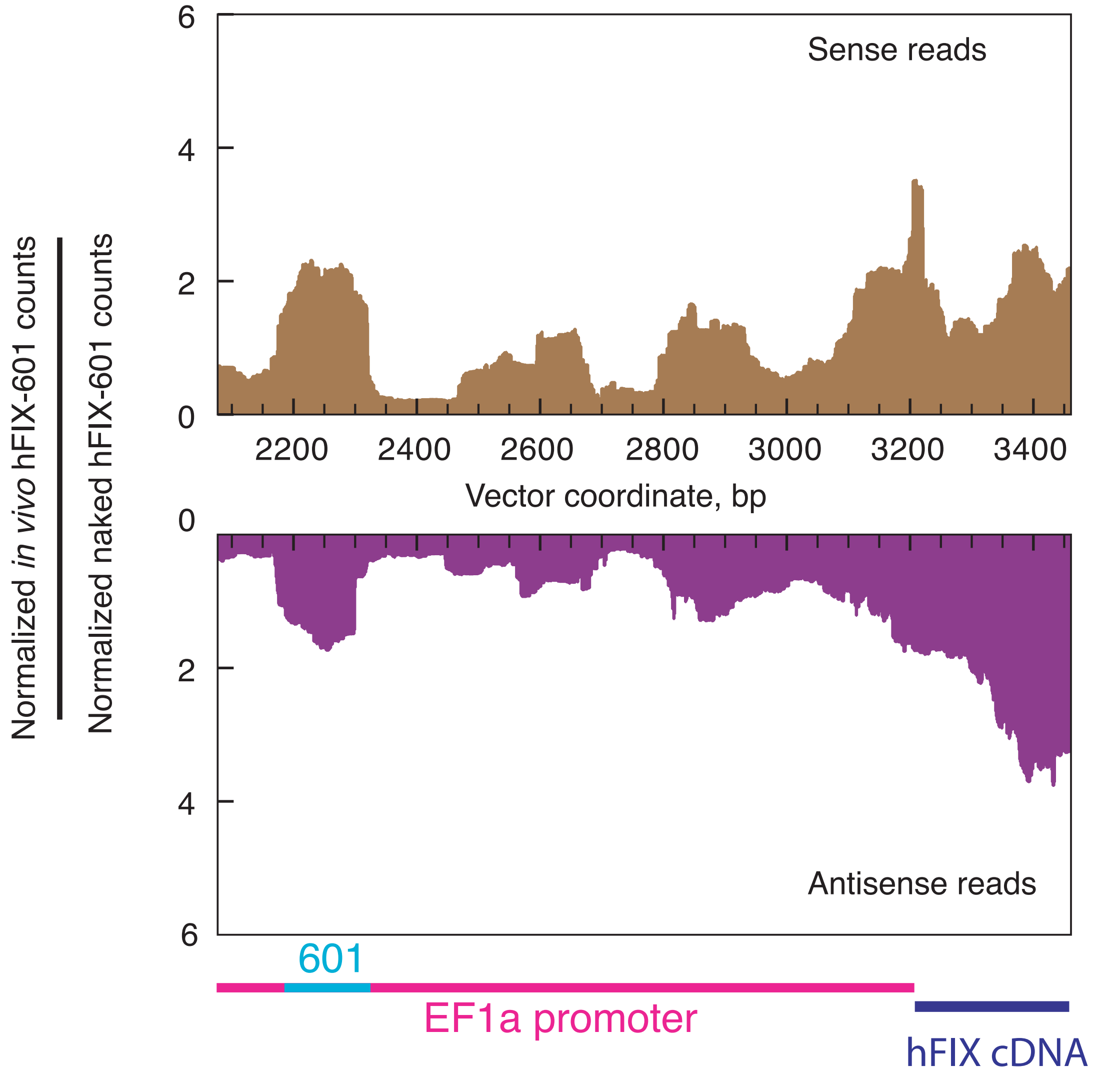

Supplementary Figure 5e

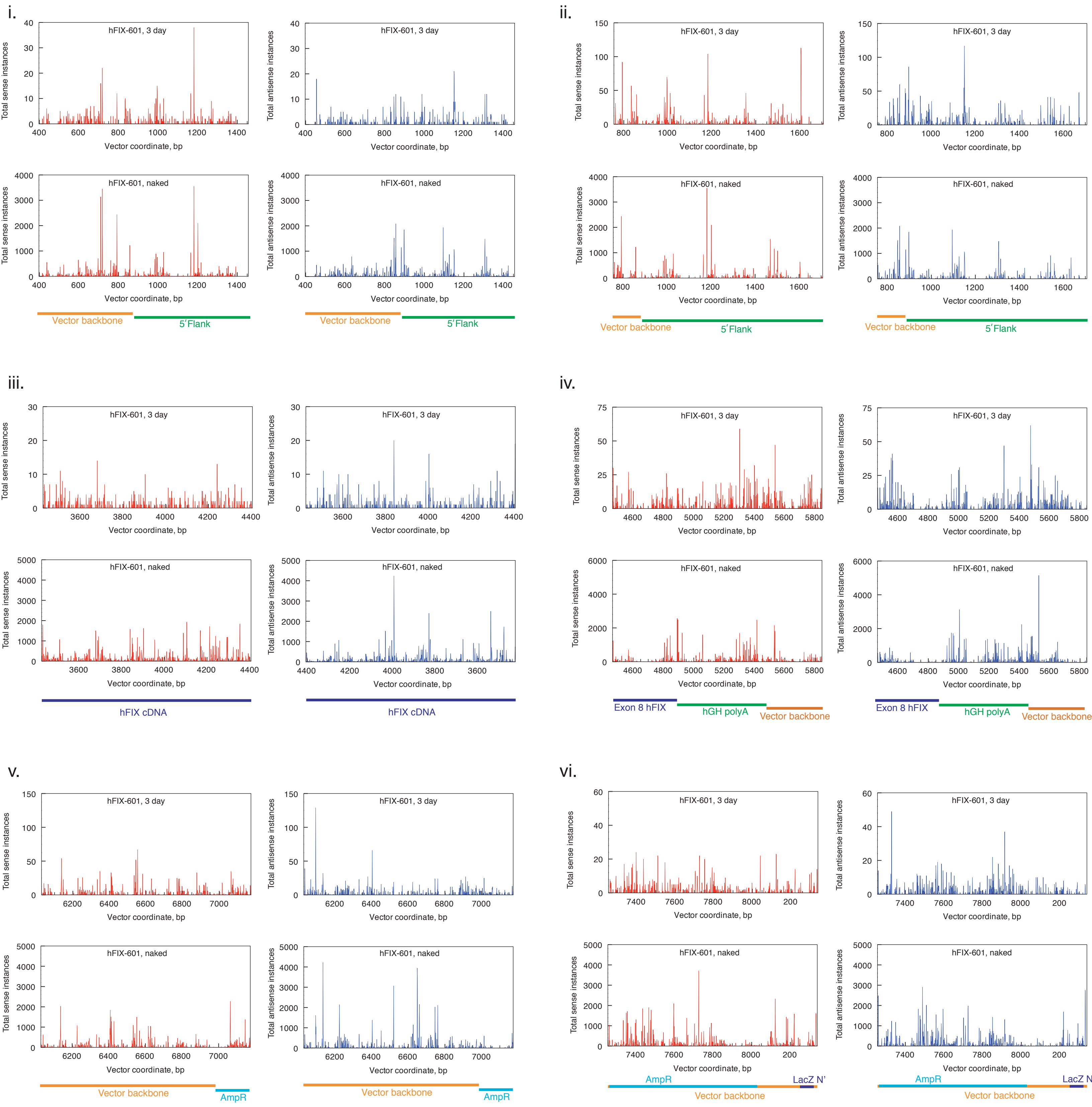

Supplementary Figure 5f

Text with Supplementary Figure 6

Since original annotations of the transcription start site (TSS) for human EF-1α were done in human cells [1], we carried out 5' RACE to check the TSS of human EF-1α (in hFIX-601) in mouse liver at 3 days post-injection. It appeared that the TSS was different in mouse liver, occurring approximately 266 basepairs downstream of the originally annotated site (Figure S6a). This updated location resulted in a larger apparent nucleosome free region (NFR) upstream of the TSS (Figure S6b).

1. Uetsuki T, Naito A, Nagata S, Kaziro Y: **Isolation and characterization of the human chromosomal gene for polypeptide chain elongation factor-1 alpha.** *J Biol Chem* 1989, **264**:5791-8.

a

69
CTTTTTCGCAACGGGTTTGCCGCCAGAACACAGGTAAGTGCCGTGTGTGGTTCCCGCCTGAGGTTTCCA
138
GTTTAAACACAAGGAGCTAGCTGGTATCCGCGGGCGGCGACGGGGCCCGTGCGTCCCAGGCACATGTT
204
CGGCGAGGCGGGGCCTGCGAGCGCGGCCACCGAGAATCGGACGGGGGTAGTCTCAAGCTGCCGGCC
266
TGCTCTGGTGCCTGGCCTCGCGCCGCCGTGTATCGCCCCGCCCTGGGCGGCAAGGCTGGCCCGGTCG

b

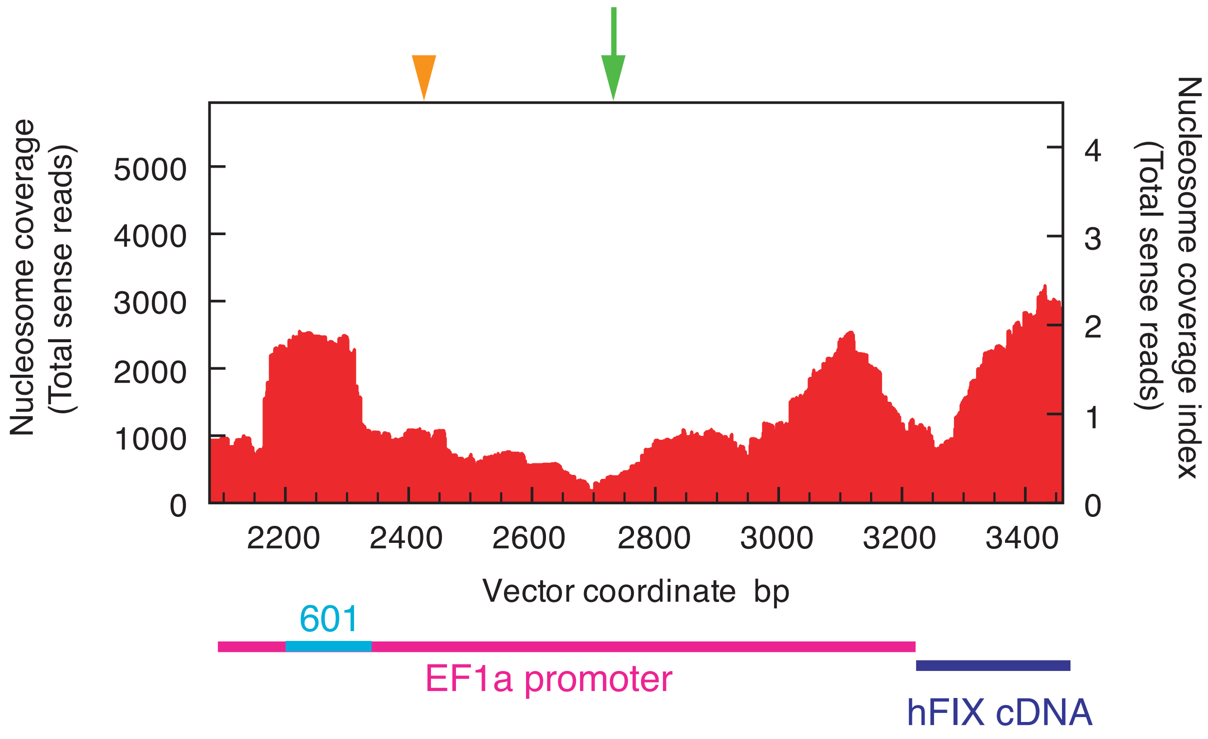

Supplementary Figure 6
